# Bridge Jobs as Paths to Working Longer: Do Gender and Retirement Identities Matter?

**DOI:** 10.1093/geroni/igaf122.120

**Published:** 2025-12-31

**Authors:** Yun Taek Oh, Phyllis Moen

**Affiliations:** University of Nevada, Reno, Reno, Nevada, United States; University of Minnesota, Minneapolis, Minnesota, United States

## Abstract

The prevalence and diversification of bridge jobs loosen the conventional coupling of older workers’ retirement and their identity transitions: not, partly, or completely retired. These transitions may vary by gender due to the pre-existing gendered norms and practices and the historical changes in women’s work lives. While these identities may influence job attachment and timing of workforce exit, the process of retirement identification, especially by gender, is not well-documented. To assess gender differences in retirement identities and timing of workforce exit of those taking on bridge jobs, we draw on a sample of Early Boomers in the Health and Retirement Study (ages 51-56 in 2004) who are in their career jobs in ages 55-56 (n = 792), and use multinomial logistic regression and event history analysis to investigate the gender differences in predictors of retirement identities and timing of workforce exit. Our results show gender differences in retirement identification; men tend to see themselves as “partly retired” (RRR = 6.201) while women tend to see themselves as “completely retired” (RRR = 5.008). Social Security receipt increases the likelihood of identifying as “partly retired” for women (RRR = 12.953) and “completely retired” for men (RRR = 30.062). Regardless of gender, self-identifying as “completely retired” is associated with earlier workforce exit while self-identifying as “not retired” is associated with longer work lives. The deep institutionalization of retirement as concomitant with Social Security receipt shapes the identities of those occupying bridge jobs, and these identities predict older workers’ workforce exits.

